# Meteorological Notices

**Published:** 1834

**Authors:** 


### XII.—Meteorological Notices.

1. The following are the results of five months' observations, by Joseph Ray, M. D., one of the teachers in the Woodward High School, in this city. They are given in continuation of those for the first quarter of the year, published in our 28th number, from the same scientific observer. The observatory is in lat. 39° 6′ 30″, and at an elevation of 500 feet above the level of the sea; north of it at a little distance, is a range of river hills rising 260 feet above its level.

TABLE.

### TABLE.

| *Thermometer* (*Fah.*) | April. | May. | June. | July. | Aug. |
|---|---|---|---|---|---|
| Mean temperature | 57.45 | 64.19 | 72.65 | 78.18 | 75.16 |
| Greatest heat | 80 | 86 | 86 | 95 | 99 |
| Least heat | 29 | 36 | 46 | 56 | 51 |
| Range | 51 | 50 | 40 | 39 | 48 |
| *Barometer.* | | | | | |
| Mean height in in. | 29.2052 | 29.2075 | 29.1761 | 29.3128 | 29.2822 |
| Greatest height | 29.86 | 29.71 | 29.411 | 29.491 | 29.41 |
| Least height | 28.68 | 28.67 | 28.899 | 29.140 | 29.12 |
| Direc. of the wind on different days | N. 6 | N. 1 | N. 15 | N. 1 | N. 2 |
| State of the weather | N.E. 6 | N.W. 13 | W. 1 | N.W. 18 | N.W. 16 |
| Depth of rain in in. | N.W. 7 | W. 1 | S.W. 9 | S.W. 8 | W. 5 |
| | W. 3 | S.W. 6 | S. 1 | S. 2 | S.W. 3 |
| | S.W. 7 | S.E. 4 | S.E. 1 | S.E. 1 | N.E. 5 |
| | S.E. 1 | N.E. 6 | N.E. 3 | N.E. 1 | |
| | 5 clear | 11 clear | 5 clear | 10 clear | 16 clear |
| | 13 cloudy | 6 cloudy | 5 cloudy | 1 cloudy | 3 cloudy |
| | 12 variable | 14 var. | 20 var. | 20 var. | 12 var. |
| | 2.83 | 2.52 | 7.64 | 3.75 | 2.42 |

2. The results which follow, are from observations, by Mr. George Mendenhall, student of medicine, and his friends, at Salem, Columbiana county, Ohio, in north latitude 40° 54′ 30″ and longitude 3° 57′ west from Washington, at a probable elevation of 800 feet above the level of the ocean. They run through the first six months of the present year, 1834.

### TABLE.

| *Thermometer.* | Jan. | Feb. | March. | April. | May. | June. |
|---|---|---|---|---|---|---|
| Mean temperature. | 24 | 42.41 | 38.15 | 53.3 | 57.52 | 68.7 |
| Greatest heat | 52 | 65 | 69 | 81 | 91 | 92 |
| Least heat | 0 | 12 | 16 | 26 | 22 | 40 |
| Range | 52 | 53 | 53 | 55 | 69 | 52 |
| Winds in days | N.W. 2 | N.W. 2 | N. 1 | N. 1 | N. 1 | N. 2 |
| | W. 6 | W. 5 | N.W. 6 | N.W. 15 | N.W. 18 | N.W. 18 |
| | S.W. 4 | S.W. 2 | W. 6 | W. 1 | W. 1 | S.W. 4 |
| | S. 2 | S. 1 | S.W. 5 | S.W. 4 | S.W. 6 | S.E. 2 |
| | S.E. 1 | S.E. 1 | S. 2 | S.E. 3 | S. 1 | |
| | E. 4 | E. 1 | E. 1 | E. 1 | S.E. 3 | |
| State of the weather | N.E. 2 | | N.E. 1 | N.E. 3 | N.E. 1 | |
| | 7 calm | 13 calm | 4 calm | 4 calm | | 2 calm |
| | 8 clear | 9 clear | 8 clear | 7 clear | | |
| | 11 cloudy | 9 cloudy | 11 cloudy | 12 cloudy | | |
| | 12 var. | 10 var. | 12 var. | 11 var. | | |

A severe frost, preceded by a strong N.W. wind, springing up in the afternoon, on the night of the 26th of April, destroyed nearly all the fruit of every kind, in the Valley of the Ohio; also a great number of annual plants on the farms and in the gardens, and the leaves and young flowers of many forest trees.

When the present calender year is through, we shall compare the observations of these two meteorologists. Meanwhile, we return or thanks to both; and take particular pleasure, in publishing an abstract of the register of Mr. Mendenhall, *because he is a student of medicine;* in the hope, that otheis will follow his example, while pursuing their studies. During that period, young gentlemen have that command of their hours, which is necessary to meteorological observations; and making them is attended with a variety of advantages to themselves:—It leads them to study the elements of meteorology, to notice the beautiful phenomena of the atmosphere, and to remark upon the influence of the various atmospheric changes on health; it promotes habits of observation, incites to punctuality, and last, though not least, makes them early risers! They should, moreover, employ themselves on this subject, from professional and patriotic considerations; for they have much in their power in these respects. If the students in the Valley of the Mississippi, for example, were generally to turn their attention to practical meteorology, they would, in a few years, collect the *data*, for a full estimate of its climate, and confer upon science and society a lasting benefit.

